# Near-Infrared Photoimmunotherapy for Oropharyngeal Cancer

**DOI:** 10.3390/cancers14225662

**Published:** 2022-11-17

**Authors:** Daisuke Nishikawa, Hidenori Suzuki, Shintaro Beppu, Hoshino Terada, Michi Sawabe, Nobuhiro Hanai

**Affiliations:** Department of Head and Neck Surgery, Aichi Cancer Center Hospital, Nagoya 464-8681, Japan

**Keywords:** oropharyngeal cancer, head and neck cancer, photoimmunotherapy, EGFR, recurrence and metastasis

## Abstract

**Simple Summary:**

Near-infrared photoimmunotherapy (NIR-PIT) represents a potential novel treatment modality for a range of cancer types, including head and neck cancers. NIR-PIT is based on the conjugation of photoactivating chemicals to cancer cell-specific antibodies. Antibody-photoabsorber-conjugate causes killing of cancer cells when activated by near-infrared light. NIR-PIT is considered to have particularly promising applications in head and neck cancers and these tumors are typically more easily accessed for illumination. Two patients with oropharyngeal lesions treated with NIR-PIT at our institution had good response with no serious adverse events and no functional disorders.

**Abstract:**

Human papillomavirus (HPV)-associated oropharyngeal cancer has a better prognosis than other head and neck cancers. However, rates of recurrence and metastasis are similar and the prognosis of recurrent or metastatic HPV-associated oropharyngeal cancer is poor. Near-infrared photoimmunotherapy (NIR-PIT) is a treatment involving administration of a photosensitizer (IRDye^®^700DX) conjugated to a monoclonal antibody followed by activation with near-infrared light illumination. It is a highly tumor-specific therapy with minimal toxicity in normal tissues. Moreover, NIR-PIT is expected to have not only direct effects on a treated lesion but also immune responses on untreated distant lesions. NIR-PIT with cetuximab-IR700 (AlluminoxTM) has been in routine clinical use since January 2021 for unresectable locally advanced or locally recurrent head and neck cancer in patients that have previously undergone radiotherapy in Japan. NIR-PIT for head and neck cancer (HN-PIT) is expected to provide a curative treatment option for the locoregional recurrent or metastatic disease after radiotherapy and surgery. This article reviews the mechanism underlying the effect of NIR-PIT and recent clinical trials of NIR-PIT for head and neck cancers, treatment-specific adverse events, combination treatment with immune checkpoint inhibitors, illumination approach and posttreatment quality of life, and provides a case of series of two patients who receive NIR-PIT for oropharyngeal cancer at our institution.

## 1. Introduction 

Head and neck cancers are the seventh most common type of cancer worldwide [1,2]. Among head and neck cancers, cancers of the oropharynx are the most common followed by cancers of the oral cavity, hypopharynx, and larynx. While most head and neck cancers are associated with smoking and alcohol, the incidence of HPV-associated oropharyngeal cancer is increasing [3,4]. HPV-associated oropharyngeal cancer differs biochemically and histopathologically from non-HPV-associated oropharyngeal cancer and is reportedly more responsive to chemotherapy and radiation therapy [5].

Although HPV-associated oropharyngeal cancer has a better prognosis than other head and neck cancers, rates of recurrence and metastasis are similar and metastatic recurrence of HPV-associated oropharyngeal cancer has a poor prognosis [6]. 

Surgery for recurrent or metastatic head and neck cancer in patients that have previously undergone radiation therapy or surgery has a high risk of serious complications including dehiscence, delayed wound healing, and carotid bleeding [7,8,9,10]. Accordingly, chemotherapy and treatment with molecularly targeted drugs or immune checkpoint inhibitors (ICI) are the current mainstays of treatment for recurrent head and neck cancer [11,12,13]. However, these therapies currently provide a very low chance of cure [14,15], even in case of locoregional disease. There is therefore an urgent clinical need for novel therapies for recurrent or metastatic head and neck cancer that can control locoregional disease without causing severe functional impairment.

Near-infrared photoimmunotherapy (NIR-PIT) was first reported in 2011 as a novel method of tumor-specific cancer treatment [16]. NIR-PIT involves administration of a light-activatable dye (IRDye^®^700DX, abbreviated to IR700) conjugated to a monoclonal antibody followed by activation with near-infrared non-thermal light. NIR-PIT is a highly tumor-specific therapy with minimal toxicity in normal tissues [17,18]. The first human phase I/IIa multicenter study of NIR-PIT with a cetuximab-IR700 conjugate (cetuximab sarotalocan sodium; RM1929) targeting epidermal growth factor receptor (EGFR) in patients with unresectable head and neck squamous cell carcinoma (HNSCC) was completed in 2017 [19]; with a “fast-tracked” global phase 3 clinical trial opening in 2019 and scheduled to be completed in 2024 (https://clinicaltrials.gov/ct2/show/NCT03769506, accessed on 12 October 2022). Although a global phase III trial is ongoing, NIR-PIT with cetuximab-IR700 (AlluminoxTM) was conditionally approved by the Pharmaceuticals and Medical Devices Agency (PMDA) of Japan in 2020 and has been in routine clinical use since January 2021 for unresectable locally advanced or locally recurrent head and neck cancer in patients that have previously undergone radiotherapy. NIR-PIT for head and neck cancer (HN-PIT) is expected to provide a curative treatment option for the locoregional recurrent or metastatic disease after radiotherapy and surgery. 

In the present review, we describe the mechanisms underlying the cytotoxicity of NIR-PIT and summarize relevant clinical studies including adverse events. In addition, we present two cases of oropharyngeal cancer treated with HN-PIT at our institution and discuss future perspectives.

## 2. Mechanism of NIR-PIT Cytotoxicity 

NIR-PIT comprises the administration of IR700, a photoactivating chemical, conjugated to monoclonal antibodies specific to a cell surface marker on cancer cells. Excitation of antibody-bound IR700 with near-infrared light at 690 nm causes IR700 to undergo a photochemical ligand reaction that releases the hydrophilic side chain of IR700 and hydrophobizes the remaining molecules. This reaction leads to damage to transmembrane target proteins thereby reducing cell membrane integrity. Damage to the cell membrane causes cancer cells to expand approximately 3-fold due to influx of extracellular fluid [20]. This rapid expansion destabilizes the cell membrane leading to the release of cytoplasmic contents. Injury to the cell membrane also leads to the activation of stress markers such as heat shock proteins 70 and 90 and pro-apoptotic signaling molecules such as calreticulin, ATP, and HMGB1 [21]. Host immune responses are then initiated against antigens released from dying cancer cells. The rapid release of cancer-specific antigens and signals induced by cell membrane damage activate local dendritic cells that then stimulate and educate cancer-specific naive T cells. Activated T cells then contribute to cell-mediated cancer cell death in a process known as immunogenic cell death (ICD) [22,23]. 

Near-infrared light is nonionizing, does not damage DNA, is harmless to normal cells, and penetrates a few centimeters into tissues [24,25]. Monoclonal antibody-photosensitive dye conjugates predominantly bind to cancer cells overexpressing cancer-associated antigens. Furthermore, IR700 is a water-soluble fluorescent molecule with negligible phototoxicity and biotoxicity [26,27] and IR700 dissociated from antibody-photoabsorber conjugate is easily excreted in urine [28]. Therefore, NIR-PIT has efficacy in selectively killing cancer cells without harming adjacent normal cells [20,29,30,31]. 

## 3. Clinical Studies 

The first clinical trials of NIR-PIT were conducted in patients with head and neck cancers for the following reasons: (1) approximately 90% of head and neck cancers have cell surface expression of EGFR; (2) light illumination of head and neck cancer sites is technically feasible; and (3) there is an urgent clinical need for novel treatments for recurrent metastatic head and neck cancer due to poor prognosis. 

A phase I/IIa, first-in-human study (RM-1929-101) of RM-1929 for the treatment of unresectable locally recurrent HNSCC was conducted in the United States in 2015 [19]. RM-1929 is a conjugate of IR700 and cetuximab, an antibody targeting EGFR which is highly expressed in HNSCC. A Japanese phase I trial (RM-1929-102) was conducted in Japanese patients with unresectable locally recurrent HNSCC in 2017 [32]. These two studies demonstrated the safety profile and efficacy RM-1929 in treating unresectable locally recurrent HNSCC. A global, multicenter, international phase III study of RM-1929 was initiated in 2018 and remains ongoing in 2022. Following the results of the RM-1929-101 and RM-1929-102 studies, RM-1929 was approved by the Japanese Ministry of Health, Labor and Welfare for the indication of “unresectable locally advanced or unresectable locally recurrent head and neck cancer” in January 2021.

### 3.1. RM-1929-101 Study 

The RM-1929-101 study was a multicenter, open-label, phase I/IIa study consisting of two parts. Part 1 was designed to determine the maximum feasible dose (MFD) of RM-1929 in patients with unresectable, locally recurrent HNSCC and to evaluate the safety profile of RM-1929. The eligibility criteria of the RM-1929-101 study were patients who could not be adequately treated with surgery, radiation, or platinum-based chemotherapy with an ECOG performance status (PS) of 0 to 2. Patients must have previously received platinum-based chemotherapy. Nine patients received 160, 320, or 640 mg/m2 of RM-1929 intravenously over 2 h on day 1 and one cycle of near-infrared light illumination on day 2 at approximately 24 h after the completion of RM-1929 administration. 

Oropharyngeal cancer was present in five out of nine patients (55.6%). All nine patients had treatment-related adverse events. The most common adverse event was pain in the treated area in three cases (33.3%). Serious adverse events (SAEs) were observed in six patients (one case each of dehydration, decreased level of consciousness, and aspiration pneumonia at 160 mg/m^2^; tumor pain and oral pain at 320 mg/m^2^; and tumor bleeding at 640 mg/m^2^). No adverse events leading to death or treatment discontinuation were observed. No dose limiting toxicity (DLT) occurred at any dose. Complete response (CR) or partial response (PR) were not observed at 160 mg/m^2^ and 320 mg/m^2^. CR was observed in one of three patients treated at a dose of 640 mg/m^2^. The mean AUC0-∞ at 640 mg/m^2^ of RM-1929 was within the range to achieve optimal EGFR saturation in tumors. Accordingly, the MFD of RM-1929 was determined as 640 mg/m^2^. 

In Part 2, 30 patients received up to a maximum of 4 cycles of 640 mg/m2 RM-1929 intravenously and light illumination at intervals of 4 to 8 weeks. Oropharyngeal carcinoma was present in seven out of 30 patients (23.3%). The median number of cycles was one cycle in 11 patients (36.7%), two cycles in seven patients (23.3%), three cycles in eight patients (26.7%), and four cycles in four patients (13.3%). SAEs occurred in 13 patients (43.3%) including three cases of pneumonia and two cases of tumor hemorrhage. Adverse events leading to death occurred in a total of 3 patients comprising tumor hemorrhage in cycle 2, arterial hemorrhage in cycle 3, and pneumonia in cycle 4. Adverse events leading to death were determined not to be related to the study treatment. Adverse events leading to discontinuation of treatment occurred in a total of five patients and included one case each of tumor hemorrhage and peripheral swelling in cycle 1, increased creatinine in cycle 2, and arterial bleeding and rash in cycle 3. Adverse events related to the study treatment were observed in 25 patients (83.3%). Major events included facial edema, fatigue, erythema, and dysphagia in five patients (16.7%), respectively, and peripheral edema, rash, tongue edema, oropharyngeal pain, and tumor pain in four patients (13.3%), respectively. 

Out of the 30 patients, four patients (13.3%) achieved CR, nine patients (30.0%) achieved PR, and 11 (36.7%) had stable disease (SD) according to the modified response evaluation criteria in solid tumors (mRECIST) ver. 1.1. The median overall survival was 9.30 months (95% confidence interval [CI] 5.16–16.92). Six-, 12- and 18-month survival rates were 63.3% (19/30), 46.7% (14/30), and 20.0% (6/30), respectively. The median progression-free survival was 5.16 months (95% CI 2.10–5.52).

### 3.2. RM-1929-102 Study 

The RM-1929-102 study was a single-center, open-label, phase I study conducted in Japan. Eligibility criteria were: patients with ECOG PS of 0 to 2 who could not be satisfactorily treated with surgery, radiotherapy, or platinum chemotherapy; and prior systemic platinum-based chemotherapy for HNSCC. This study comprised three patients administered 640 mg/m^2^ of RM-1929 intravenously over 2 h on day 1 and one cycle of near-infrared light illumination on day 2 approximately 24 h after the completion of RM-1929 administration. Oropharyngeal carcinoma was present in one of three patients (33.3%). Adverse events were observed in all three cases (100%), with 13 adverse events (pain in the treated area, edema, glossitis, liver enzyme elevation, hypertension, and increased gamma-glutamyl transferase) determined to be related to the study treatment. No SAEs, deaths, or adverse events leading to treatment discontinuation were observed. No DLTs were observed. Two of the three patients achieved PR and one patient had disease progression (PD) according to mRECIST ver. 1.1. 

## 4. Treatment Flow of HN-PIT in Clinical Practice

### 4.1. Day 1: Administration of RM-1929 

On day 1 or the day of RM-1929 administration, the environment around the patient was prepared for RM-1929 administration. Patients were asked to avoid direct sunlight and room illuminance was kept below 120 lux. RM-1929 was administered at a dose of 640 mg/m^2^ by intravenous infusion over 2 h. 

### 4.2. Day 2: Laser Illumination 

**Figure 1:**
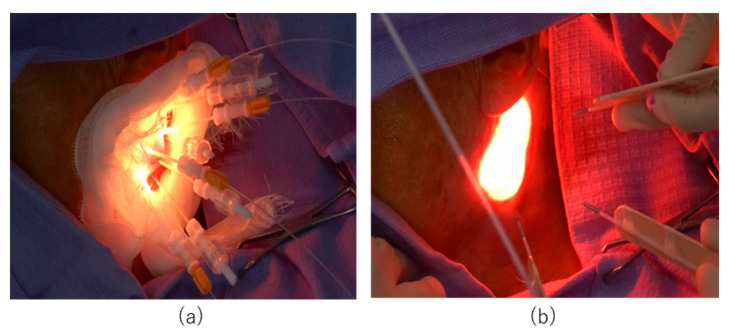
Laser illuminating devices. (**a**) Near-infrared laser illumination using cylindrical diffusers for a deep or thick lesion. (**b**) Near-infrared laser illumination using frontal diffusers for a superficial lesion.

At 20–28 h after completion of the RM-1929 infusion, near-infrared laser illumination was performed using the BioBlade^®^ laser system (Rakuten Medical, Inc., San Diego, CA, USA). Laser diffusers were selected according to tumor location. For deep lesions (tumors deeper than 10 mm below the skin or mucosal surface) or thick lesions (tumors thicker than 10 mm), a cylindrical diffuser was used for laser illumination (Figure 1a). Superficial lesions (tumors located within 10 mm from the skin or mucosal surface) were treated using a frontal diffuser (Figure 1b). 

When using a cylindrical diffuser, a needle catheter was first inserted into the tumor. If tumors were large in diameter, multiple needle catheters were inserted at a maximum distance of 18 mm to avoid overlap with the illuminated area, thus allowing the laser beam to illuminate the entire tumor. A cylindrical diffuser was inserted into the lumen of the needle catheter to illuminate the laser beam. When treating with a frontal diffuser, the area to be illuminated was first determined including a margin of at least 5 mm. The minimum diameter of the laser beam that can be emitted by a single frontal diffuser is 17 mm and the maximum diameter is 38 mm. If the planned illumination area was larger than 38 mm, multiple frontal diffusers were used or illumination was divided into multiple sessions. The two types of diffusers were used in combination according to lesion location and size. Subsequent treatment cycles were performed up to a maximum of four cycles spaced at least four weeks apart. 

## 5. Treatment-Specific Adverse Events 

Patients become photosensitive after RM-1929 administration because as EGFR is also expressed by normal skin and mucosal cells [33,34,35,36]. Therefore, appropriate measures to protect against light exposure should be taken. The environment around patients should be maintained below 120 lux for one week after the administration of RM-1929. Patients should avoid skin exposure when in brighter areas. Sun exposure should be avoided for four weeks after the administration of RM-1929. The incidence of photosensitivity in the RM-1929-101 study was 3/39 patients (7.7%), with grade 1 photosensitivity in two patients and grade 2 photosensitivity in one patient. This incidence of photosensitivity is considerably lower than the severity and frequency of photosensitivity associated with photodynamic therapy [37]. Other adverse events requiring caution include carotid and tumor hemorrhage, tongue edema and laryngeal edema, infusion reaction, severe skin reactions, and hypomagnesemia. HN-PIT causes rapid tumor collapse which can lead to fatal bleeding after treatment in patients with tumor invasion of large blood vessels. Therefore, the treatment of tumors with carotid invasion is contraindicated due to the risk of carotid rupture. Severe edema can develop after HN-PIT due to increased levels of reactive oxygen species generated by the treatment [17,38]. As laryngeal edema leads to airway obstruction, tracheostomy should be performed prior to light illumination when treating areas close to the airway.

## 6. Treatment Indications 

HN-PIT, NIR-PIT for head and neck cancer, is indicated in Japan for unresectable locally recurrent or locally advanced head and neck cancer with prior radiotherapy. In other words, HN-PIT is considered appropriate for the treatment of unresectable tumors only. Common reasons for unresectable head and neck cancer include carotid artery invasion, extensive skull base invasion, and mediastinal or vertebral invasion. However, the use of HN-PIT is not indicated for the treatment of these lesions. Therefore, the lesion must be determined to be unresectable based on other factors. In our previous study of HN-PIT, tumors were considered unresectable for the following reasons: (1) the high risk of further surgery or reconstructive surgery; (2) the decreased likelihood of complete cure after further surgery due to multiple previous resections; (3) poor general condition limiting reconstructive surgery; and (4) patient refusal of further surgery despite clinical recommendations. 

Decisions regarding treatment options are often difficult in cases of locoregional recurrence of oropharyngeal lesions that have previously undergone reconstructive surgery or radiation. Additional open surgery is high risk and transoral resection is often technically challenging due to changes in anatomical structure. HN-PIT is a tumor-specific treatment that has demonstrated efficacy and less damage to normal tissue even in patients that have previously undergone multiple treatments. 

## 7. Case Presentation 

Ten patients underwent a total of 19 cycles of HN-PIT at Aichi Cancer Center Hospital (Table 1). Herein, we have described two patients of oropharyngeal lesions treated with HN-PIT. Tumor response evaluation was conducted using modified RECIST 1.1 [19].

### 7.1. Case 1 

**Figure 2:**
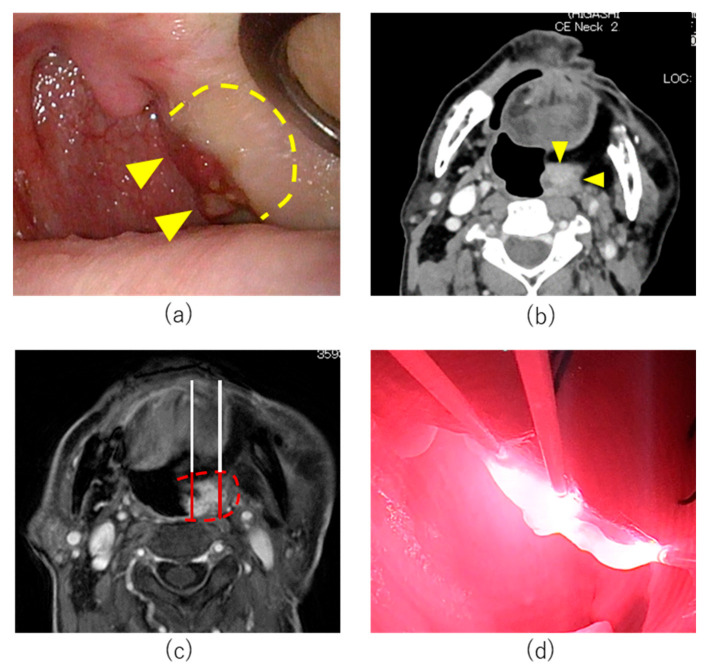
Treatment course of Case 1. (**a**) Pretreatment endoscopic findings: Left oropharyngeal tumor (arrowheads) and tumor induration (dotted line). (**b**) Axial section of Gd-enhanced MRI T1 weighted image. (**c**) Treatment plan of cycle 1 HN-PIT treatment. Planned illumination area (dotted line). (**d**) Intraoperative image of HN-PIT in Cycle 1. Near-infrared laser was illuminated using 20 mm cylindrical diffusers.

An 80-year-old man had undergone partial tongue resection and radiotherapy for tongue cancer 40 years previously. Maxillary, oropharyngeal, and mandibular resection, left neck dissection, and free rectus abdominis musculocutaneous flap reconstruction for mandibular gingival carcinoma T4aN0M0 (UICC 8th edition) had been performed one year previously. Local recurrence in the lateral wall of the oropharynx developed two months prior to attending our institution. Endoscopy demonstrated a lesion on the dorsal side of the musculocutaneous flap of the lateral wall of the left oropharynx (Figure 2a). No carotid infiltration was observed (Figure 2b). The tumor was 14 mm in anterior-posterior diameter and 34 mm in superior-inferior diameter. Pharyngocutaneous fistulation was considered unlikely due to the distance between the tumor and the skin surface. The tumor was considered unresectable for the following reasons. Transoral resection was not considered possible as it was difficult to resect the appropriate layer due to previous surgery. Open surgery for resection and reconstruction was considered high risk. Other treatment options including chemotherapy and ICI were excluded as they were not curative treatments. Accordingly, HN-PIT was selected in this case.

On day 1, RM-1929 was administered without complications. On day 2, tracheostomy was performed under general anesthesia prior to laser illumination. The pharynx was exposed using a Feyh-Kastenbauer Weinstein-O’Malley (FK-WO) retractor (Olympus, Tokyo, Japan). The tumor location and border were confirmed using ultrasonography. A treatment margin of 5 mm was set around the entire circumference of the tumor and six needle catheters were inserted under ultrasonography (SonoSite SII, Fujifilm, Tokyo, Japan) and endoscopy (ENDOEYE FLEX, Olympus, Tokyo, Japan) (Figure 2c). Laser illumination was performed with cylindrical diffusers of 20 mm length (Figure 2d). 

**Figure 3:**
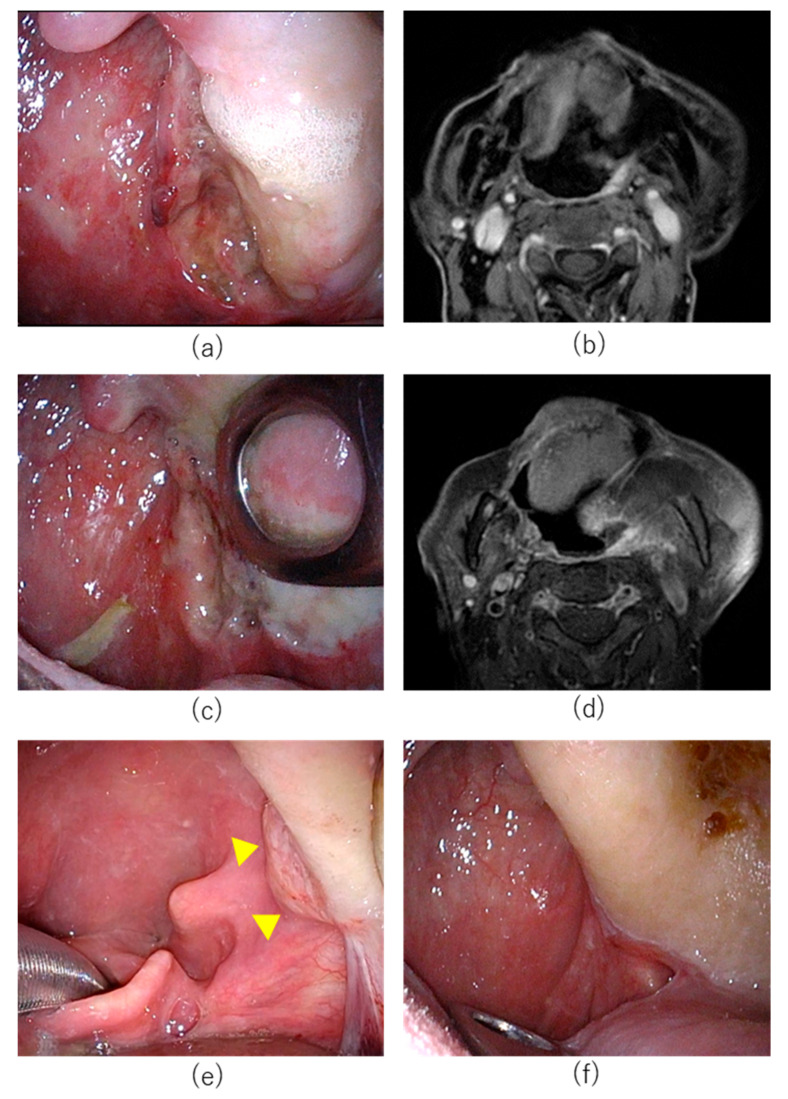
Posttreatment change after HN-PIT for Case 1. (**a**) Endoscopic image on postoperative day 9 after cycle 1. Ulceration was observed in the illuminated area. (**b**) MRI image after cycle 1. (**c**) Endoscopic image on postoperative day 6 after cycle 2. (**d**) MRI image after cycle 2. (**e**) Intraoperative image of the tumor (arrowheads) before transoral resection. (**f**) endoscopic image on postoperative 4 months.

Continuous intravenous fentanyl was administered intraoperatively for prophylaxis of postoperative pain. No postoperative pain, edema, or bleeding was observed. On postoperative day (POD) 1, fentanyl was stopped and tube feeding was initiated through a previously created gastrostomy. The patient was discharged on POD 14. On POD 9, the tumor had almost disappeared with necrotic ulceration observed (Figure 3a). On POD 23, the ulcer had shrunk and tissue granulation had increased. However, biopsy of the granulated area revealed residual tumor cells. Magnetic resonance imaging (MRI) on POD 21 demonstrated the tumor size had decreased compared to pretreatment images (Figure 3b). As biopsy confirmed residual tumor cells, a second round HN-PIT was administered. In an attempt to increase treatment effect, an increased number of diffusers were used and seven needle catheters were inserted. The postoperative course of the second cycle was similar to cycle 1. Although the tracheostomy was closed at the patient’s request after cycle 1, pharyngolaryngeal edema did not occur after illumination in cycle 2. Postoperative pain was mild and fentanyl was not used. Rapid tumor shrinkage was again observed (Figure 3c). The patient was discharged on POD 7 after cycle 2. MRI imaging on POD 27 demonstrated further decreases in tumor size; however, residual tumor was again suspected (Figure 3d). We therefore recommended a third round of HN-PIT; however, the patient declined further treatment with HN-PIT and instead elected to be carefully followed up without further treatment. Four months after the second round of PIT, a biopsy of granulation tissue revealed residual tumor cells (Figure 3e). As the size of the tumor had decreased significantly, we considered transoral resection to be feasible at this time. Accordingly, transoral resection was performed five months after the second cycle of HN-PIT. The tumor was resected from the middle of the pharyngeal constrictor muscle. No scarring or wound healing complications were observed. Pathological analysis of the surgical specimen demonstrated that the oropharyngeal lesion had differing histological features from the original maxillary gingival carcinoma. Additionally, immunohistochemical staining revealed that p16 was negative. Therefore, the oropharyngeal lesion was considered a new p16-negative oropharyngeal carcinoma rather than recurrence of the original maxillary gingival carcinoma. No recurrence was observed at 16 months postoperatively and the patient remains under observation (Figure 3f). 

### 7.2. Case 2 

**Figure 4:**
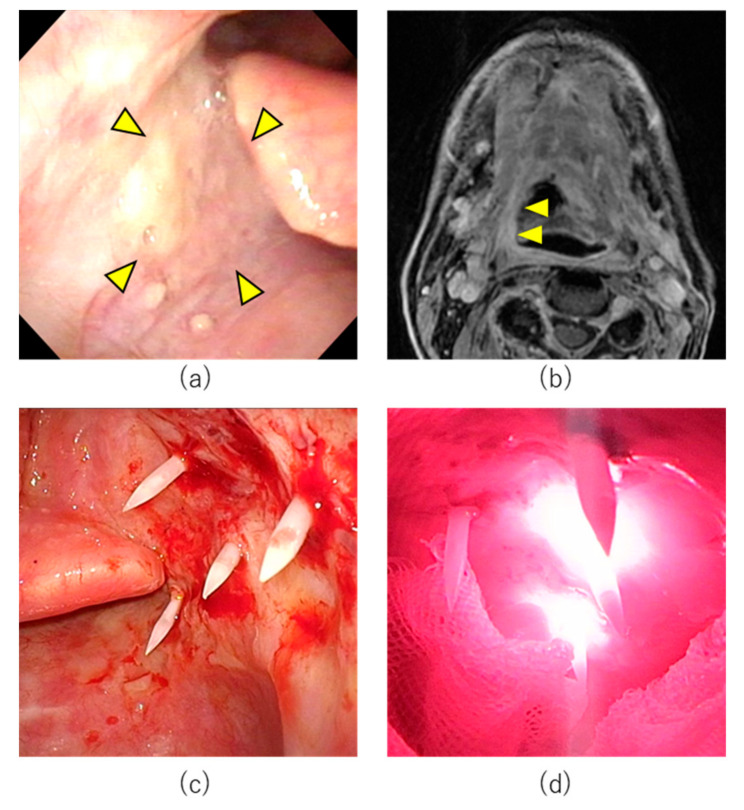
Treatment course of Case 2. (**a**) Pretreatment endoscopic findings with white light. Right base of tongue tumor (arrowheads). (**b**) Axial section of Gd-enhanced MRI T1 weighted image. The tumor was enhanced (arrowheads). (**c**) Needle catheters were punctured percutaneously into the pharynx in HN-PIT. (**d**) Near-infrared laser illuminated the tumor.

A 77-year-old male patient had undergone concurrent chemoradiotherapy after neck dissection for hypopharyngeal cancer 6 years previously. Transoral resection for cancer of the base of tongue had been attempted one year previously; however, the tumor had been resected using a mandibular swing approach as exposure of the tumor had been technically challenging using a retractor. In the same year, he had undergone transoral resection of soft palate cancer. Subsequently, cancer of the base of tongue (SCC, p16 negative, cT1N0M0 UICC 8th edition) was detected and the patient was referred to Aichi Cancer Center Hospital from another hospital (Figure 4). The tumor was located from the base of the tongue to the vallecula. Although the tumor diameter was unmeasurable on CT and MRI, based on the endoscopic findings, we determined that the tumor was a superficial lesion of <20 mm in diameter. It did not involve the carotid or lingual arteries. The tumor was considered unresectable for the following reasons. Regarding transoral resection, pharyngeal exposure was considered technically challenging. The risk of open surgery for resection was considered high due to previous surgery. As chemotherapy and ICI were not curative treatments, HN-PIT was selected in this case. 

On day 1, the patient received intravenous RM-1929 with no complications. On day 2, tracheostomy was first performed under general anesthesia followed by pharyngeal exposure with an FK-WO retractor. The tumor was a superficial tumor located on the right base of tongue. Near-infrared light must be directed perpendicularly to the lesion when using a frontal diffuser. As vertical illumination for this area was technically challenging, we selected laser illumination with cylindrical diffusers. Percutaneous insertion of four needle catheters and laser illumination with cylindrical diffusers of 20 mm length was performed under endoscopy and ultrasonography. Near-infrared laser illumination was then performed. 

**Figure 5:**
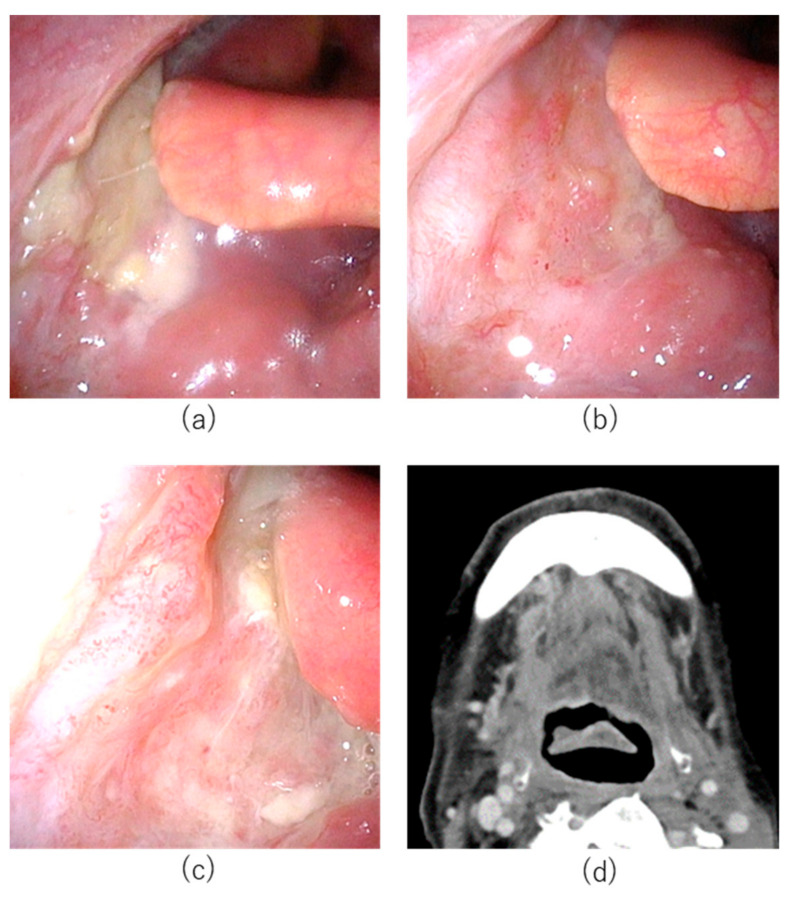
Posttreatment change after HN-ALX for Case 2. (**a**) Endoscopic image on postoperative day 21 after cycle 1. White coating was observed on the illuminated area. (**b**) Endoscopic image on postoperative day 98 after cycle 1. The mucosa was almost normalized with no obvious tumor. (**c**) Endoscopic image 11 months after cycle 2. The mucosal surface was slightly irregular. Biopsy did not detect evidence of cancer. (**d**) CT image 11 months after cycle 2. No tumor mass was observed.

The patient had postoperative pain which was treated with intravenous administration of fentanyl, non-steroidal anti-inflammatory drugs (NSAIDs), and acetaminophen. Severe pain had resolved by POD 1 and fentanyl administration was discontinued. Edema of the neck, face, and larynx was observed on POD 1 but had almost completely resolved by POD 6. No other complications were observed and the patient was discharged on POD 7. Although CR could not be confirmed, the tracheal cannula was removed one month postoperatively as the patient declined a second cycle of HN-PIT. Eleven months after cycle 2, the mucosal surface appeared slightly irregular. However, biopsy did not reveal obvious residual cancer cells. The patient remains under observation with no obvious recurrence at 13 months after the first cycle of HN-PIT (Figure 5).

## 8. Immune Activation

As mentioned above, NIR-PIT has been posited to cause ICD that promotes maturation of immature dendritic cells in the cancer microenvironment [21]. Newly primed CD8+ T cells after NIR-PIT have been shown to response to a greater range of cancer antigens [39]. Although NIR-PIT may not kill all cancer cells immediately, activation of host immunity may lead to the clearance of a high proportion of remaining tumor cells. Conventional systemic cancer immunotherapies include T cell activating type 1 cytokines (e.g., IL-2 and IL-15) and ICI (e.g., anti-CTLA4 and anti-PD1/PDL1 antibodies). These therapies work on the principle of activating existing CD8+ T cells and, therefore, may induce autoimmune disease [40]. Unlike these therapies, NIR-PIT enhances host immunity locally without systemic adverse events.

NIR-PIT selectively eliminates immunosuppressive cells in the local tumor environment when used with antibodies against CD25 or CCR4 on the surface of Treg cells and CXCR2 on the surface of MDSCs [41]. Depletion of local Treg cells using Treg-targeted NIR-PIT against CD25 was highly effective in a syngeneic mouse model [42]. CD8+ T cells and NK cells in the treated tumor bed were fully activated within hours of Treg depletion by NIR-PIT. Treg-targeted NIR-PIT also affected non-treated tumors despite the treatment being directed at a single target lesion. When targeting CD25 systemically, activated effector cells expressing CD25 are also depleted. Furthermore, systemic blockade of immunosuppressive regulatory mechanisms may induce adverse autoimmune events. On the other hand, Treg-targeted NIR-PIT, which selectively depletes intra-tumoral Treg cells, avoids systemic adverse effects.

## 9. Combined Treatment with ICI 

The use of NIR-PIT alone has been largely unable to induce sustained antitumor responses in syngeneic tumor mouse models. Adaptive immune resistance may limit sustained responses after treatment with NIR-PIT. The combination of NIR-PIT with an anti-PD-1 inhibitor has been shown to reverse adaptive immune resistance and enhance preexisting tumor antigen-specific T cell responses and de novo T cell responses induced by NIR-PIT in multiple allogeneic tumor models [39]. Combination therapy led to complete rejection of tumors treated with NIR-PIT and untreated distant tumors. Tumor antigen-specific T cell responses were observed in both treated and untreated tumors, demonstrating the development of systemic antitumor immunity. Although this strategy has demonstrated efficacy in animal models, this approach may have undesirable side effects associated with the use of ICI. The combination of NIR-PIT and ICI resulted in rapid tumor shrinkage within a few days of treatment. Even after tumor removal, the immune system of treated animals was able to reject tumors after re-inoculation, indicating the persistence of systemic immune memory of the initial tumor. A clinical study of NIR-PIT in combination with ICI for HNSCC and cutaneous squamous cell carcinoma is currently ongoing (ClinicalTrials.gov identifier: NCT04305795 accessed on 12 October 2022).

## 10. Laser Illumination Approach

NIR-PIT requires precise illumination of target lesions with near-infrared light, which is a major factor affecting efficacy and safety. Treatment is performed using a combination of a cylindrical diffusers to illuminate deep or thick lesions or a frontal diffuser to illuminate superficial lesions. Although the head and neck region is an easier area to illuminate than other parts of a body, accurate illumination of deep lesions under the skin and mucosa and lesions deep in the upper aerodigestive tract, such as hypopharynx, is technically challenging. In the present cases, needle catheter insertion was performed under endoscopic and ultrasound guidance after pharyngeal exposure with a retractor and illumination was performed with cylindrical diffusers. There have been several reports of HN-PIT using imaging modalities. Omura et al. treated a nasopharyngeal lesion with a frontal diffuser under rigid endoscope assistance [43]. Okamoto et al. illuminated a lesion within the lateral pterygoid muscle with cylindrical diffusers and performed needle catheter insertion using the Surgical Navigation System [44]. Koyama et al. reported needle catheter insetion and cylindrical diffuser illumination of a lesion in the maxillary sinus, which was not visible from the outside, using the Surgical Navigation System and CT guidance [45]. Thus, laser illumination must be performed precisely using a combination of imaging modalities.

However, there are areas where illumination is technically challenging even with use of the above imaging modalities, such as the hypopharynx. As the frontal diffuser is only able to illuminate lesions in front of the light socket, there is a need for the development of diffusers that are able to illuminate lesions in the lateral wall of the upper aerodigestive system.

## 11. Impact on Quality of Life

In patients with unresectable head and neck cancer, quality of life (QOL) can be compromised before and after treatment [46]. The progression of tumors in the head and neck region can result in marked functional decline in swallowing, speech, and breathing as well as symptoms of severe pain and bleeding [47]. Previous health-related QOL studies in patients with head and neck cancers have shown that QOL is particularly affected by oropharyngeal cancer, including increasing the risk of malnutrition [48]. In addition, conventional treatments of unresectable head and neck cancer after radiation include chemotherapy and ICI, which may reduce QOL due to treatment-related adverse events [11,12,13]. Anticipated functional declines due to treatment have been shown to influence treatment choice [49]. In HN-PIT, adverse events are predominantly localized while systemic adverse events are rare. Therefore, HN-PIT may represent a promising treatment option for unresectable head and neck cancer. Okamoto et al. assessed functional scales (physical, role, emotional, cognitive, and social functioning) and global health status as well as domain scales (pain, swallowing, sense problems, speech problems, trouble with social eating, trouble with social contact, and reduced sexuality) using the EORTC QLQ-C30 and QLQ-H&N35 questionnaires, with no significant changes in any of the QOL endpoints reported after HN-PIT [50]. Head and neck cancer survivors may suffer from treatment sequelae over a long period of time even in cases of CR to treatment [51]. HN-PIT may contribute to the maintenance of QOL after treatment.

## 12. Conclusions

We report the treatment of two patients with oropharyngeal lesions at our institution. Treatment responses were good in both cases with no severe adverse events and no functional disorders observed after HN-PIT. At present, HN-PIT is a predominantly local treatment. However, future approaches combining HN-PIT with ICI or Treg-targeting NIR-PIT may also have efficacy in the treatment of distant metastatic disease.

## Figures and Tables

**Table 1:** Characteristics of patients treated with HN-PIT.

Case	Gender	Age	ECOG PS	Histology	Primary site	Location of Target Lesion	Diffuser	Cycle	Complication	BOR
1	M	84	1	SCC	Floor of mouth	Cervical skin	Cylindrical, frontal	3	Pain G2 Bleeding G2 Edema G1	PR
2	M	84	1	SCC	Upper gingiva	Oropharynx	Cylindrical	2	Pain G1	PR
3	M	54	0	SCC	Upper gingiva	Subcutaneous tissue of face	Cylindrical	2	Pain G2 Edema G1 Fistula G1	CR
4	M	77	0	SCC	Oropharynx	Oropharynx	Cylindrical	1	Pain G2 Edema G1	PR
5	M	68	0	SCC	Larynx	Glottis	Cylindrical, frontal	3	Edema G1	PR
6	M	79	1	SCC	Oropharynx	Cervical skin	Cylindrical, frontal	2	Pain G2	PR
7	M	42	0	SCC	Buccal mucosa	Tongue	Cylindrical	1	Pain G2 Edema G2	CR
8	M	88	1	SCC	Lower gingiva	Lower gingiva	Cylindrical, frontal	1	Edema G4	CR
9	F	74	1	SCC	Maxilla	Nasal cavity	Cylindrical	3	Pain G1	PR
10	M	80	1	SCC	Oral cavity	Subcutaneous tissue of face	Cylindrical	1	Fistula G2	PR

ECOG-PS, Eastern Cooperative Oncology Group Performance Status; BOR, best overall response; SCC, squamous cell carcinoma; PR, partial response; CR, complete response.

## Data Availability

Not applicable.
